# DIFFERENTIAL DIAGNOSIS BETWEEN BILIARY AND NONBILIARY ACUTE PANCREATITIS: WHAT IS THE IMPORTANCE OF LABORATORY TESTS?

**DOI:** 10.1590/0102-672020220002e1694

**Published:** 2022-11-25

**Authors:** Barbra Rafaela de Melo Santos Azevedo, Djalma José Fagundes

**Affiliations:** 1 Municipal Hospital Dr. José de Carvalho Florence, General Surgery, São José dos Campos – São Paulo (SP), Brazil;; 2 Universidade Federal de São Paulo, Operative Technique and Experimental Surgery – São Paulo (SP), Brazil.

**Keywords:** Pancreatitis, Differential Diagnosis, Biochemistry, Pancreatite, Diagnóstico Diferencial, Bioquímica

## Abstract

**BACKGROUND:**

The differential diagnosis of the causal factors of acute pancreatitis is fundamental for its clinical follow-up, becoming relevant to establishing laboratory criteria that elucidate the difference between biliary and nonbiliary causes.

**AIM:**

The aim of this study was to establish criteria based on laboratory tests for the differential diagnosis between acute pancreatitis of biliary and nonbiliary causes and to identify laboratory tests with sufficient sensitivity to propose the creation of an algorithm for differential diagnosis between the causes.

**METHODS:**

The research consisted of observational analysis, with a cross-sectional design of laboratory tests of two groups of patients with acute pancreatitis: group A: nonbiliary cause and group B: biliary cause. Hematocrit, white blood cell count, lactate dehydrogenase, glucose, lipase, amylase, total bilirubin, oxalacetic transaminase, pyruvic transaminase, gamma-glutamyltransferase, and alkaline phosphatase were investigated. Data were submitted to nonparametric tests and receiver operating characteristics.

**RESULTS:**

Hematocrit values, number of leukocytes, lactate dehydrogenase, and glucose showed no significant difference between the groups (p>0.1). Lipase, amylase, total bilirubin, oxalacetic transaminase, pyruvic transaminase, gamma-glutamyltransferase, and alkaline phosphatase values showed a significant difference between groups (p<0.05). The oxalacetic transaminase, pyruvic transaminase, and alkaline phosphatase tests were most sensitive in determining the biliary cause, allowing the establishment of a cutoff point by the receiver operating characteristic test: pyruvic transaminase: 123.0 U/L (sensitivity: 69.2%; specificity: 81.5%), oxalacetic transaminase: 123.5 U/L (sensitivity: 57.3%; specificity: 78.8%), and alkaline phosphatase: 126.5 U/L (sensitivity: 66.1%; specificity: 69.4%), from which the probability of a correct answer increases.

**CONCLUSION:**

It was possible to establish criteria based on laboratory tests for the differential diagnosis between acute pancreatitis of biliary and nonbiliary origin; however, the tests did not show enough sensitivity to propose the creation of an algorithm for differential diagnosis between the same causes.

## RESUMO

**RACIONAL:**

O diagnóstico diferencial dos fatores causais da pancreatite aguda é fundamental para seu seguimento clínico, tornando-se relevante estabelecer critérios laboratoriais que elucidem a diferença entre as causas biliares e não biliares.

**OBJETIVOS:**

Estabelecer critérios baseados em testes laboratoriais para o diagnóstico diferencial entre pancreatite aguda de causa biliar e não biliar e identificar testes laboratoriais com sensibilidade suficiente para propor a criação de um algoritmo de diagnóstico diferencial entre as causas.

**MÉTODO:**

Análise observacional, com delineamento transversal, de exames laboratoriais de dois grupos de pacientes com pancreatite aguda: A — causa não biliar; e B — causa biliar. Foram investigados: hematócrito, número de leucócitos, lactato desidrogenase, glicose, lipase, amilase, bilirrubina total, transaminase oxalacética, transaminase pirúvica, gamaglutamiltransferase e fosfatase alcalina. Os dados foram submetidos a testes não paramétricos e ao *receiver operating characteristic.*

**RESULTADOS:**

Os valores de hematócrito, número de leucócitos, lactato desidrogenase e glicose não apresentaram diferença significante entre os grupos (p>0.1). Os valores de lipase, amilase, bilirrubina total, transaminase oxalacética, transaminase pirúvica, gamaglutamiltransferase e fosfatase alcalina apresentaram diferença significante entre os grupos (p<0.05), sendo que os testes de transaminase oxalacética, transaminase pirúvica e fosfatase alcalina mostraram-se os mais sensíveis na determinação da causa biliar, possibilitando o estabelecimento de um ponto de corte pelo teste *receiver operating characteristic*, a partir do qual a probabilidade de acerto aumenta: transaminase pirúvica: 123,0 U/L (sensibilidade: 69,2%; especificidade: 81,5%), transaminase oxalacética: 123,5 U/L (sensibilidade: 57,3%; especificidade: 78,8%) e fosfatase alcalina: 126,5 U/L (sensibilidade: 66,1%; especificidade: 69,4%).

**CONCLUSÃO:**

Foi possível estabelecer critérios baseados em testes laboratoriais para o diagnóstico diferencial entre pancreatite aguda de origem biliar e não biliar, porém, os testes não mostraram sensibilidade suficiente para propor a criação de um algoritmo de diagnóstico diferencial entre as mesmas causas.

## INTRODUCTION

Acute pancreatitis (AP) is among the most common gastrointestinal manifestations for which hospitalization is mandatory, generating a significant impact on health services, in terms of management and costs^8,16,25,29^.

The obstruction of the pancreatic duct by gallstones seems to be one of the main processes responsible for this mechanism, since it induces an increase in ductal pressure, generating an accumulation of enzyme-rich fluid in the organ tissue^9,16^.

Prolonged use of alcohol constitutes the second cause of AP, and, in most cases, the induction of an outbreak overlaps with a preestablished condition of chronic pancreatitis, which may generate severe incapacitating pain^8,9,13^.

The diagnosis of AP, by the Atlanta Classification (USA), reviewed in 2012, requires the presence of at least two of the three criteria:

1. abdominal pain consistent with the diagnostic hypothesis;
2. serum amylase and/or lipase values of at least three times the upper limit of normality; and
3. suggestive findings on imaging examinations, such as contrast-enhanced CT and/or abdominal ultrasound^1,4,8,9,16,29,30^.

The establishment of laboratory markers as predictors of the differential diagnosis of AP has already been proposed in studies, such as the existence of a probable relationship between lipase and amylase values to differentiate the biliary cause from the alcoholic cause, but there are no results considered to be in consensus^11,26^. Other tests, such as total bilirubin, alkaline phosphatase, pyruvic transaminase, oxalacetic transaminase, and even amylase and lipase, in isolation, seem to be able to determine the origin of AP, with pyruvic transaminase being pointed out as having the most significant positive predictive value in defining the biliary origin^6,9,11,15,17,23,26,31,32^.

The first widely used AP severity scale dates back to 1974, with the publication of the Ranson Criteria (modified in 1982), which can estimate the morbidity and mortality related to the condition^7,20^. Although several other scores have been proposed and some authors still consider the Ranson Criteria to be limited, especially since they require 48 h to be defined^1,2,16^, they are still widely used owing to their easy application and specificity in determining the prognosis of the disease^24^.

Considering that the differential diagnosis of the causal factors of AP is essential for its treatment and clinical follow-up, it becomes relevant to establish criteria that clarify the difference between biliary and nonbiliary causes upon patient admission to the hospital. Establishing criteria based on laboratory tests for the differential diagnosis may be an advantage to the use of imaging tests since the laboratory tests are more available and less expensive when compared to the imaging ones.

This research aimed to establish criteria based on laboratory tests for the differential diagnosis between AP of biliary and nonbiliary causes and to identify laboratory tests with sufficient sensitivity to propose the creation of an algorithm for differential diagnosis between the causes.

## METHODS

The research consisted of observational analysis, with a cross-sectional design, of patients at Municipal Hospital Dr. José de Carvalho Florence (HMJCF), in São José dos Campos, São Paulo. This is a public hospital, which provides services to patients exclusively through the Brazilian Public Health System (Sistema Único de Saúde — SUS). The research received approval from the Research Ethics Committee of the Federal University of São Paulo (protocol no. 1059/2019), as well as authorization from the HMJCF for its execution. Since it was based on the analysis of electronic databases, the Research Ethics Committee (REC) waived the need for an informed consent form. For data collection, a list of medical care records between January 2014 and December 2018 was requested from the HMJCF Medical Records and Statistics Service, based on the *International Classification of Diseases* (*ICD*), including codes corresponding to AP diagnoses, as follows:

- K85.0 — acute idiopathic pancreatitis
- K85.1 — acute biliary pancreatitis
- K85.2 — alcohol-induced acute pancreatitis
- K85.3 — drug-induced acute pancreatitis
- K85.8 — other acute pancreatitis
- K85.9 — unspecified acute pancreatitis
- K86.0 — alcohol-induced chronic pancreatitis
- K86.1 — other chronic pancreatitis
- K86.3 — other specified pancreas diseases
- K86.9 — pancreas disease not otherwise specified
- K87.1 — pancreas disorders in diseases elsewhere classified

Patients over 18 years were included in the study, and all were admitted to HMJCF under the aforementioned codes.

The selected patients were distributed into groups according to the causative agent of pancreatitis, based on consulting the discharge or death summaries available in the electronic database of patients seen and admitted to HMJCF.

Patients were divided into two groups:

- Group A: patients diagnosed with nonbiliary AP.
- Group B: patients diagnosed with biliary AP.

Since this is a study considering patients included within the same population (the same hospital, under similar conditions of care and treatment), convenience sampling was applied.

The diagnosis of the patients was established based on the information entered by the attending physicians in the discharge or death summaries and further confirmation through laboratory tests. The biliary etiology was confirmed by abdominal ultrasonography, performed by a single device, Acuson NX3 Elite model from Siemens, and by three experienced radiologists, showing gallstones, and the nonbiliary etiology was determined by excluding the presence of gallstones in the same examination.

After identifying the groups, the laboratory tests requested at the time of hospital admission were analyzed, and only the results recorded within 48 h of the first request were considered.

Considering the proposed comparison between the groups, the results of the following laboratory parameters were analyzed:

- Hematocrit — reference value: 39–50%.
- White blood cell count — reference value: 3500–10,500 mm^3^.
- Lactate dehydrogenase (LDH) — reference value: 313–618 U/L.
- Glucose — reference value (fasting): 70–99 mg/dl.
- Lipase — reference value: 23–300 U/L.
- Amylase — reference value: 30–110 U/L.
- Total bilirubin — reference value: 0.2–1.3 mg/dl.
- Oxalacetic transaminase (GOT) — reference value: 14–36 U/L.
- Pyruvic transaminase (GPT) — reference value: 9–52 U/L.
- Gamma-glutamyltransferase (GGT) — reference value: 12–43 U/L.
- Alkaline phosphatase (ALP) — reference value: 38–126 U/L.

The selected biochemical tests were chosen based on what the literature points out as the ones that present the greatest variations in the differential diagnosis of AP^6,9,11,15,17,23,26,31,32^, in addition to the tests considered for establishing Ranson's severity criteria^7,20^.

Patients whose data did not include the measurement of serum lipase or did not have a value of at least three times the upper limit of normality, considering the test that confirms in laboratory the diagnosis of AP more specifically, were excluded from the study^1,4,8,9,16,25,29,30^.

Although, by the Atlanta Classification, the presence of two of the three criteria is sufficient to establish a diagnosis of AP^25^, it was decided to make the lipase test mandatory, since the reports of the imaging examinations were not fully recorded in the electronic database of the HMJCF.

The statistical difference of the results between the two groups was analyzed using the Stata^®^ software, and the Shapiro-Wilk test was applied to evaluate the normality of the distribution, and afterward, the Kruskal-Wallis test was used for two independent groups. In cases where “p” was less than 0.05, the statistical difference was considered significant, and when “p” was greater than 0.1, the difference was considered nonsignificant, where “p” is the probability of erroneously concluding by significance.

To evaluate the accuracy of the diagnostic tests, analyzing their sensitivity and specificity, the receiver operating characteristic (ROC) analysis was applied, expressed by the corresponding curves and the area under the curve (AUC) values, using the SPSS^®^ software.

The AUC analysis provides an estimate of the overall accuracy of the test, and its value may be interpreted as follows: poor (0.5–0.6), bad (0.6–0.7), poor (0.7–0.8), good (0.8–0.9), or excellent (>9), according to the performance in predicting the parameter evaluated^19^.

All the data collected in the research are stored in a password-protected file on the personal computer of the researcher in charge and will be kept for at least 5 years after the end of the work.

## RESULTS

In the patient selection, several causes were found for AP, highlighting the biliary (571–61.73%), alcoholic (149–16.11%), and drug (129–13.95%). Among the causes with lower occurrences are those of idiopathic nature (22–2.38%); those caused by surgical acts involving the pancreas and bile ducts (19–2.05%), neoplastic diseases (16–1.73%), hypertriglyceridemia (10–1.08%), and, less commonly, abdominal trauma (5–0.54%); and those that occurred after endoscopic retrograde cholangiopancreatography (ERCP) (4–0.43%).

Of the 925 patients eligible for the study, 224 were excluded due to either not recording a lipase value or because their value was not equal to or greater than three times the laboratory's reference limit as preestablished.

**Figure 1:**
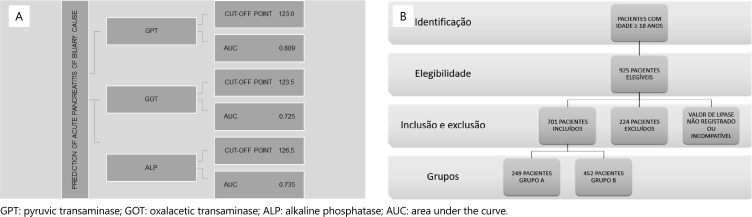
(A) Prediction of acute pancreatitis of biliary cause. (B) Flow diagram of study participant selection.

Therefore, 701 patients were included in the study: 249 (36%) were diagnosed with nonbiliary AP, included in group A, and 452 (64%) were diagnosed with biliary AP, included in group B. Figure 1A shows the prediction of acute pancreatitis of biliary cause and Figure 1B shows the flow diagram of study participant selection.

The statistical analysis concluded that hematocrit, leukocyte count, LDH, and glucose values showed no significant difference between groups A and B. Table 1 shows the medians and “p” values of the different laboratory tests investigated, which were not statistically significant.

**Table 1:** Median results of hematocrit, white blood cell count, lactate dehydrogenase, and glucose tests for the 701 patients in group A (nonbiliary acute pancreatitis) and group B (biliary acute pancreatitis). Without statistical significance by Shapiro-Wilk and Kruskal-Wallis tests (p>0.1).

Comparison between the medians of groups A and B			
Test	Group A	Group B	p-value
Hematocrit	40.9	41	0.5205
Number of leukocytes	11.600	11.440	0.6487
LDH	619	611	0.8989
Glycose	98	97.5	0.7433

LDH: lactate dehydrogenase.

When comparing the results of the tests applied between groups A and B, a statistically significant difference was found between the values of lipase, amylase, total bilirubin, GOT, GPT, GGT, and ALP, all of which were higher in group B than in group A. Table 2 shows the medians and “p” values of the measurements of the different laboratory tests investigated, which were significant.

**Table 2:** Median results of lipase, amylase, total bilirubin, oxalacetic transaminase, pyruvic transaminase, gamma-glutamyltransferase, and alkaline phosphatase tests for the 701 patients in group A (nonbiliary acute pancreatitis) and group B (biliary acute pancreatitis). Statistical significance was shown by Shapiro-Wilk and Kruskal-Wallis tests (p<0.05).

Comparison between the medians of groups A and B				
Test	Group A	Group B	p-value	Test
Lipase		3.210	6317.5	0.0001
Amylase		556.5	1.043	0.0001
Total bilirubin		1.2	2.05	0.0001
GOT		59	180	0.0001
GPT		52	252	0.0001
GGT		193	432	0.0001
ALP		110.5	174	0.0001

GOT: oxalacetic transaminase; GPT: pyruvic transaminase; GGT: gamma-glutamyltransferase; ALP: alkaline phosphatase.

**Figure 2:**
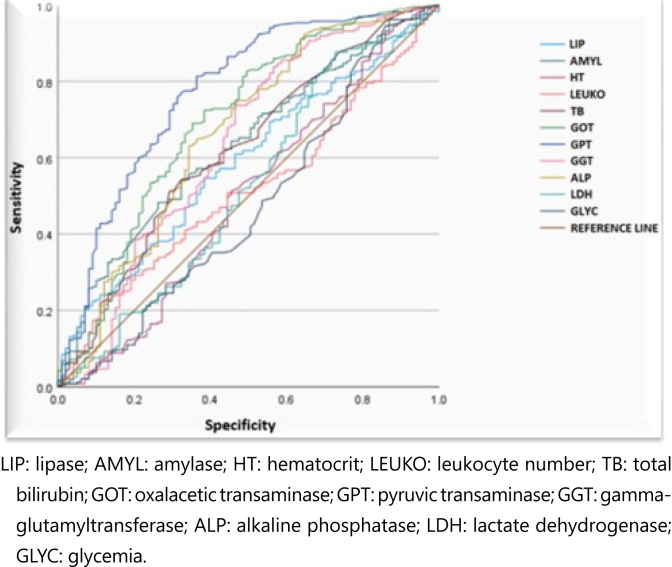
Receiver operating characteristics curve representing the accuracy of the biochemical tests evaluated in predicting the differential diagnosis of biliary and nonbiliary acute pancreatitis by analyzing sensitivity and specificity.

The ROC result obtained for the analyzed biochemical tests showed great variation in the accuracy of their performance as predictors of the differential diagnosis of AP (Table 3 and Figure 2).

**Table 3:** Data obtained through receiver operating characteristics analysis of the biochemical tests of oxalacetic transaminase, pyruvic transaminase, and alkaline phosphatase in predicting the diagnosis of acute biliary pancreatitis (group B), considering only values up to the ninth decile.

	GPT	GOP	ALP
n	523.0	538.0	440.0
Standard deviation	150.42	119.55	65.53
Q1	43.0	41.0	89.0
Median	115.0	101.0	128.0
Q3	259.0	207.0	182.5

Q1: first quartile; Q3: third quartile; GPT: pyruvic transaminase; GOP: oxalacetic transaminase; ALP: alkaline phosphatase.

**Figure 3:**
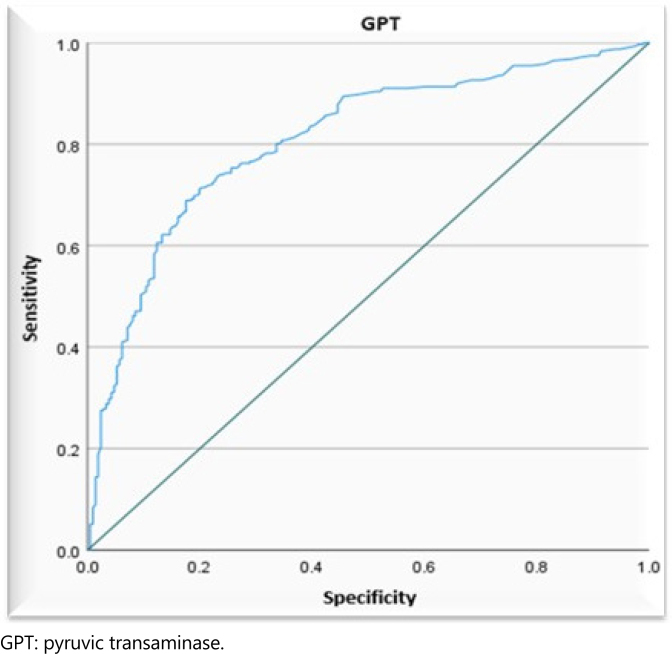
Receiver operating characteristics curve, demonstrating the accuracy of the individual pyruvic transaminase biochemical test in predicting the biliary cause of acute pancreatitis, analyzing the sensitivity and specificity, considering values up to the ninth decile.

**Figure 4:**
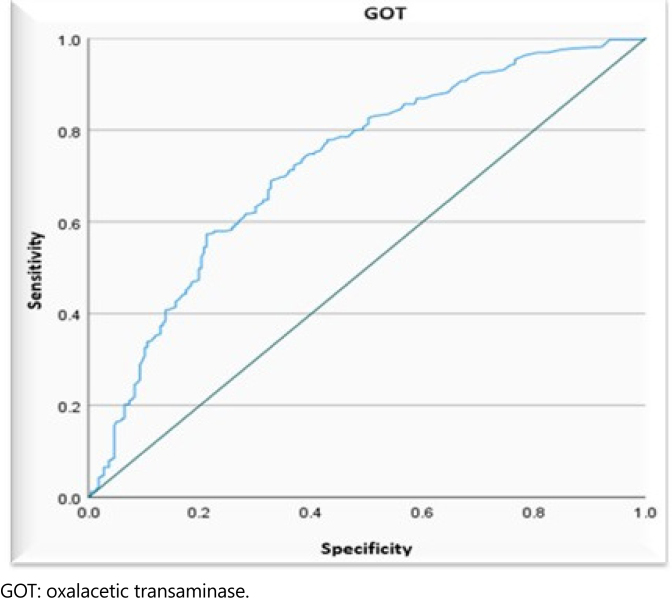
Receiver operating characteristics curve, demonstrating the accuracy of the biochemical test of oxalacetic transaminase, individually, in predicting the biliary cause of acute pancreatitis, analyzing their sensitivity and specificity, considering values up to the ninth decile.

**Figure 5:**
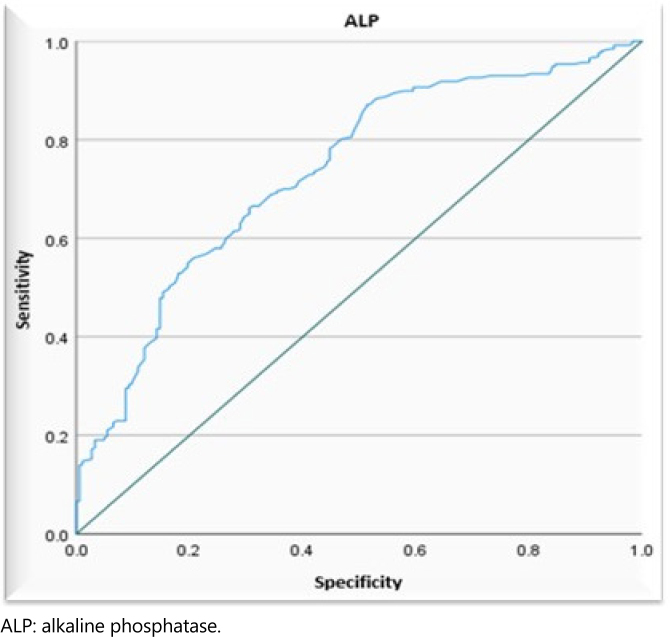
Receiver operating characteristics curve, demonstrating the accuracy of individual alkaline phosphatase biochemical tests in predicting the biliary cause of acute pancreatitis, analyzing their sensitivity and specificity, considering values up to the ninth decile.

The ROC analysis applied individually showed greater importance of GPT, GOT, and ALP tests as predictors for the PA diagnosis of biliary etiology. However, these tests had discrepant values (outliers) concerning the median of the groups, and the values were considered only up to the ninth decile (Figures 2–5).

By analyzing the ROC curve and the AUC, it was possible to establish cutoff points from which the cause of AP would probably be of biliary origin, as well as to estimate the sensitivity and specificity of the tests for this purpose (Table 4).

**Table 4:** Receiver operating characteristics curve and area under the curve analysis demonstrating the sensitivity and specificity of the biochemical tests of pyruvic transaminase, oxalacetic transaminase, and alkaline phosphatase in predicting the diagnosis of acute biliary pancreatitis (Group B), with their respective cutoff points, considering values up to the ninth decile.

	Cutoff points	Sensitivity (%)	Specificity (%)	AUC
GPT	123.0	69.2	81.5	0.809
GOT	123.5	57.3	78.8	0.725
ALP	126.5	66.1	69.4	0.735

AUC: area under the curve; GPT: pyruvic transaminase; GOT: oxalacetic transaminase; ALP: alkaline phosphatase.

Some patients did not have complete laboratory data records, evidencing the number of tests that were not requested within the first 48 h of admission or were not in the hospital's laboratory analysis system (Table 5).

**Table 5:** Number of patients and laboratory tests according to biliary (B) and nonbiliary (A) etiology groups for acute pancreatitis.

Total number of patients selected for the study = 701		
Test	Biliary = 452	Nonbiliary = 249
Lipase	452	249
Amylase	443	248
Hematocrit	450	249
Number of leukocytes	450	249
Total bilirubin	408	212
GOT	433	243
GPT	423	237
GGT	363	215
ALP	350	206
LDH	374	166
Glycose	354	147

GOT: oxalacetic transaminase; GPT: pyruvic transaminase; GGT: gamma-glutamyltransferase; ALP: alkaline phosphatase; LDH: lactate dehydrogenase.

## DISCUSSION

Promptly directing specific treatment to the etiology of AP, after the initial evaluation, provides a better prognosis, as early differential diagnosis influences subsequent therapeutic interventions, modifying the course of the disease and significantly decreasing morbidity and mortality^4,9,15,17,21,23,24,29^.

Numerous studies have sought to establish laboratory criteria for the differential diagnosis between biliary and nonbiliary AP, especially alcoholic AP. Other reports recognize the importance of this definition. Nevertheless, most of them have not reached a consensus to establish a reliable score due to variability of results or the limited number of samples, especially the national studies^6,9,11,15,17,23,27,31,32^.

In this study, when a possible relationship between the values of the laboratory tests chosen as representative of the disease and the biliary and nonbiliary etiology of AP was analyzed, the results obtained showed a similar association with the literature.

The increased hematocrit rate, although it may be related to the severity and worse prognosis of the disease^3,14,17,28^, did not have representative values in the diagnostic elucidation of AP in our sample. No studies were found in the literature that pointed to the number of leukocytes as a viable variable to differentiate the causal factor of AP.

The LDH is well established as a predictor of severity for AP^5,22^, although it is not pointed out as a diagnostic marker, as is blood glucose. However, increased glucose values are mentioned as indicators of poor prognosis for AP^12,18^.

Table 1 shows the values of the hematocrit, white blood cell count, LDH, and blood glucose tests, which, similar to the literature findings, showed no significant difference between the groups.

Trying to establish a correlation between the increased lipase values and the differential diagnosis of AP has been the objective of previous studies, without a consensus^10,23^. Increased amylase values, in contrast, are often associated with AP of biliary cause^4,6,9,15^. Our results demonstrate that lipase and amylase values in the biliary PA group were higher than in the nonbiliary PA group.

There are divergences as to the applicability of total bilirubin dosage in the diagnosis of AP,^16,26,31,32^ and several studies have already confirmed a relationship between the increase in hepatic transaminases and the biliary cause of AP, with GPT being the best established biochemical test as the most sensitive marker, with high positive predictive value^15,17,27^.

The results of this study showed higher values of total bilirubin, GOT, and GPT in the biliary PA group compared to the nonbiliary PA group.

The canalicular enzymes, ALP and GGT, are also indicated as predictors of biliary etiology in AP, although normal values do not exclude the diagnosis^6,17,31,32^. In this study, ALP and GGT showed higher values in the biliary PA group than in the nonbiliary PA group.

Although the tests showed a statistically significant difference between the values of lipase, amylase, total bilirubin, GOP, GPT, GGT, and ALP (Table 2) when comparing the biliary and nonbiliary AP groups, a predominance of the biliary AP group in all data, when the ROC analysis, a more robust tool in the validation of diagnostic tests, was performed, it did not corroborate most of them as positive predictors for AP of biliary origin.

Among the tests indicated by the Shapiro-Wilk and Kruskal-Wallis tests as statistically significant for the differential diagnosis of AP, only GPT, GOT, and ALP presented values with sufficient sensitivity and specificity for the definition of a cutoff point, from which the probability of a biliary cause for AP is higher.

The determination of the cutoff points, as well as the AUC, for GPT, GOT, and ALP was made through tables generated by the SPSS^®^ software, based on the interpretation of the ROC curves created for each test.

A relevant factor seems to have contributed to the fact that the tests did not show satisfactory accuracy in the ROC analysis: some patients had incomplete laboratory data, which shows the number of tests that were not requested in the first 48 h of hospitalization or were not in the database entries (Table 5).

Another contributing factor to the decreased performance of the tests analyzed as predictors for the differential diagnosis of AP was the existence of outliers concerning the median of the groups. To minimize this occurrence, we adopted test values only up to the ninth decile, i.e., 90% of the GPT, GOT, and ALP tests (Table 3 and Figures 3–5).

It is noteworthy that most probably the number of patients found in the database is underestimated, since most pancreatitis may have been recorded under a less specific *ICD*, such as “abdominal pain,” for example, making the search unfeasible. In addition, the patients, who were excluded for not having the value of lipase reported, probably had external tests, requested in the Emergency Care Unit (ECU), before admission to the HMJCF, which is the reference hospital in the region.

Despite the limiting values for sensitivity in determining the cause of AP, for the reasons presented, it was possible to establish cutoff points for GPT (123.0 U/L), GOT (123.5 U/L), and ALP (126.5 U/L), from which the disease would most likely have a biliary origin (Table 4).

Since it is considered the test with the highest positive predictive value in defining the cause of pancreatitis, the GPT (ALT) already has a cutoff point well established by some studies as ≥150.0 U/L for biliary origin^15,17,27^. In this study, the value at which the biliary cause was most likely considered was GPT ≥123.0 U/L (Table 4).

The GPT dosage was also the test with the highest AUC (0.809), confirming its accuracy in determining the biliary origin of AP, which is considered a reliable test for this purpose, according to this classification (Table 4).

The observational and statistical analysis of the data obtained in this study allows the creation of a protocol based on laboratory criteria, so that the cause of AP can be defined by simple and rapid tests.

The sample size and the sensitivity of the tests were limiting factors inherent to the database researched. Given the data collected, it was not possible to establish how large the study population should be and what degree of sensitivity the tests should have to be considered adequate substitutes for the current diagnostic criteria.

## CONCLUSION

It was possible to establish criteria based on laboratory tests for the differential diagnosis between AP of biliary and nonbiliary origin; however, the tests did not show sufficient sensitivity to propose the creation of an algorithm for differential diagnosis between the two.
